# The role of magnetic resonance imaging in Ménière disease: the current state of endolymphatic hydrops evaluation

**DOI:** 10.31744/einstein_journal/2019MD4743

**Published:** 2019-02-19

**Authors:** Rafael Maffei Loureiro, Daniel Vaccaro Sumi, Marcelo Delboni Lemos, Hugo Luis de Vasconcelos Chambi Tames, Regina Lucia Elia Gomes, Mauro Miguel Daniel, Carolina Ribeiro Soares, Rodrigo Watanabe Murakoshi, Marcelo Buarque de Gusmão Funari

**Affiliations:** 1 Hospital Israelita Albert Einstein, São Paulo, SP, Brazil.

**Keywords:** Magnetic resonance imaging, Ménière disease, Labyrinth diseases, Saccule and utricle, Ear, inner, Endolymphatic hydrops, Imagem por ressonância magnética, Doença de Ménière, Doenças do labirinto, Sáculo e utrículo, Orelha interna, Hidropsia endolinfática

## Abstract

Technical advances in magnetic resonance imaging have allowed to accurately detect and grade endolymphatic space distension in Ménière disease; this was only possible in *post-mortem* histological studies until a few years ago. Magnetic resonance imaging rules out other causes of vertigo and hearing loss, and is able to evaluate the cochlear and vestibular compartments of the endolymphatic space using a dedicated protocol.

## RESUMO

Os avanços técnicos na ressonância magnética têm permitido detectar e classificar com acurácia a distensão do espaço endolinfático na doença de Ménière; isso só era possível nos estudos histológicos *post-mortem* até poucos anos atrás. Além de afastar outras causas de vertigem e de perda auditiva, a ressonância magnética é capaz de avaliar os compartimentos coclear e vestibular do espaço endolinfático por meio de um protocolo dedicado.

## INTRODUCTION

Ménière disease (MD) is a clinical syndrome of unknown etiology, and consists of intermittent episodes of vertigo, often associated with fluctuating sensorineural hearing loss, tinnitus and aural fullness.^(^ ^1^ ^)^ The pathogenesis of MD is attributed to endolymphatic hydrops (EH), characterized by distension of the labyrinthine structures that contain endolymph: cochlear duct, saccule, utricle, ampullae and semicircular ducts, corroborated by *post-mortem* studies.^(^ ^2^ ^)^ However, the relation between MD and EH is complex and not yet fully established.

Only recently has magnetic resonance imaging (MRI) been able to detect EH in MD, allowing its confirmation *in vivo*. Nakashima et al.,^(^ ^3^ ^)^ demonstrated endolymphatic space distension in patients with MD using a three-dimensional fluid-attenuated inversion recovery (3D-FLAIR) sequence, in a 3-Tesla field device, 24 hours after intratympanic administration of gadolinium. As gadolinium accumulates in the perilymph and does not reach the endolymph, it is possible to differentiate these two compartments and to demonstrate EH.

Since then, new protocols and sequences have been developed to differentiate the endolymphatic and perilymphatic compartments in clinical practice, and many of them use intravenous gadolinium administration. Although the intratympanic route results in a higher concentration of gadolinium in perilymph, it consists of an off-label use, is less practical and requires a 24-hour wait before image acquisition. On the other hand, the intravenous route has the following advantages: it is less invasive, evaluates both ears at the same time, does not depend on the permeability of the oval and round windows, allows to evaluate the blood-labyrinth barrier and requires a shorter waiting time before image acquisition (4 hours).^(^ ^4^ ^)^ Three-dimensional fluid-attenuated inversion recovery and inversion recovery turbo spin echo with real reconstruction (3D real-IR) are among the most used MRI sequences to characterize the signal differences between perilymph (with contrast) and endolymph (without contrast).

**Figure 1:**
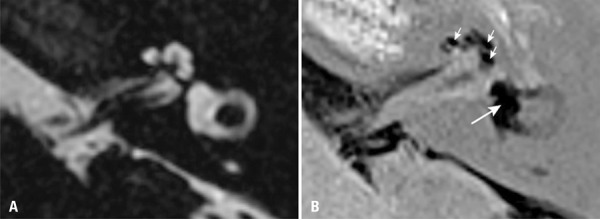
Axial magnetic resonance imaging of the left ear of a patient diagnosed with Ménière disease. (A) Highly T2-weighted sequence shows the labyrinthine fluid space (sum of endolymphatic and perilymphatic spaces). (B) Inversion recovery turbo spin echo with real reconstruction (3D real-IR) sequence 4 hours after intravenous gadolinium administration shows distension of saccule and utricle, which occupy most of the vestibular area (long arrow), and significant distension of cochlear duct (short arrows)

Regarding EH grading, one of the first methods used was the one proposed by Nakashima et al*.,* ^(^ ^5^ ^)^ which classified EH as none, mild and significant according to the ratio between the endolymphatic space and the labyrinthine fluid space (sum of endolymphatic and perilymphatic spaces). Vestibular hydrops would be considered mild if the endolymphatic space occupied between 33.3% and 50% of the vestibular fluid space, or significant if it occupied more than 50% (Figure 1). Cochlear hydrops would be considered mild if the dilated cochlear duct was smaller than the vestibular scale, or significant if the cochlear duct was larger than the vestibular scale (Figure 1). However, some authors have questioned this method by demonstrating that the area of endolymphatic space can be altered according to the inversion time used in image acquisition.^(^ ^6^ ^,^ ^7^ ^)^ Other semiquantitative graduation methods were described using different values of cut-off and evaluation criteria, but there is still no consensus in the literature about the method to be used.^(^ ^4^ ^)^

A recent meta-analysis demonstrated an order of EH progression in MD, which begins in the cochlea and then involves the saccule, utricle, ampullae and finally the semicircular ducts.^(^ ^2^ ^)^ In addition, the degree of distension of the membranous labyrinth structures appears to be related to its mechanical complacency, which is high in the saccule and lower in the utricle and semicircular ducts.^(^ ^8^ ^)^ In this context, Attyé et al*.,* ^(^ ^7^ ^)^ described a new criterion for EH evaluation called SURI (inversion of the saccule to utricle area ratio), which was only observed in patients with MD (sensitivity: 50% and specificity: 100%).

Several studies have shown the relation between EH detection and grading in MRI with the clinical findings in patients with MD.^(^ ^1^ ^,^ ^9^ ^,^ ^10^ ^)^ In one of them,^(^ ^9^ ^)^ 90% of patients with MD had endolymphatic hydrops in MRI, similar frequency found in histopathological studies. In addition, the progression of EH over time and the correlation with loss of cochlear and vestibular function in patients with MD have been demonstrated.^(^ ^1^ ^,^ ^10^ ^)^ These studies also revealed the presence of variable EH in the asymptomatic ear of patients with unilateral clinical presentation, indicating that MD can be a systemic disease with bilateral evolution over time.

The MRI advances have shown that this imaging method is a robust tool in the evaluation of EH, with results similar to those found in *post-mortem* temporal bone studies. Magnetic resonance imaging allows to rule out other causes of vertigo and hearing loss, such as vestibular schwannomas, and also to evaluate separately the cochlear and vestibular compartments of the endolymphatic space with a dedicated protocol. The imaging acquisition and evaluation techniques of EH are still under development, but it is possible that new large-scale studies validate MRI as an accurate tool in the diagnostic criteria of MD in a near future.
